# Four-Year Changes in Visceral Fat Mass and the Risk of Developing Proteinuria in the General Population

**DOI:** 10.1371/journal.pone.0131119

**Published:** 2015-06-17

**Authors:** Jwa-Kyung Kim, Young-Jun Kwon, Young Rim Song, Young-Su Kim, Hyung Jik Kim, Sung Gyun Kim, Young-Su Ju

**Affiliations:** 1 Department of Internal Medicine, Hallym University Sacred Heart Hospital, Kidney Research Institute, Anyang, Korea; 2 Department of Occupational and Environmental Medicine, Hallym University Sacred Heart Hospital, Anyang, Korea; Graduate School of Medicine, Osaka University, JAPAN

## Abstract

**Background:**

Previous cross-sectional studies demonstrated the close relationship between visceral obesity and the increased prevalence of proteinuria. But, little is known about the role of changes in visceral fat mass (∆VFM) over several years in the development of proteinuria. In this longitudinal cohort study with the general population, the changes in ∆VFM as well as baseline VFM on proteinuria development were evaluated.

**Methods:**

Healthy individuals (n = 2393) who participated in two health screening exams were analyzed. Subjects were divided into three groups based on gender-specific tertiles of baseline VFM and ∆VFM. Each patient was tested for proteinuria using a dipstick, and proteinuria was defined as 1+ or greater.

**Results:**

The mean age was 51.9±7.7 years, and the incidence of proteinuria was 3.9% (n = 93). During the 4 years, 52.5% of the subjects experienced a decline in ∆VFM. However, subjects who developed proteinuria exhibited a significant increase in ∆VFM. Even after adjustment for age, smoking, systolic and diastolic BP, serum creatinine, and hs-CRP levels, the highest tertiles for baseline VFM [men, odds ratio (OR) 3.43, 95% confidence interval (CI) 1.22–9.67; women, OR 2.01, 95% CI 1.05–4.15] and ∆VFM (men, OR 2.92, 95% CI 1.22–6.99; women, OR 3.16, 95% CI 1.56–6.39) were independent predictors of proteinuria development. Following adjustment of both parameters, subjects in the highest baseline VFM and ∆VFM tertiles exhibited the greatest risk of proteinuria development, which suggested the additive harmful effects of the two factors.

**Conclusions:**

Baseline VFM and greater increase in ∆VFM were both important risk factors for developing proteinuria in the general population. Appropriate education and interventions to prevent accumulation of VFM should be the major focus of preemptive strategies.

## Introduction

As the prevalence of obesity continues to increase, the theory that obesity is a chronic, inflammatory, and contagious disease has become widely accepted. To date, numerous studies have demonstrated the close relationship between obesity and various conditions with high morbidity and mortality, such as coronary heart disease, ischemic stroke, type 2 diabetes, cancer, and osteoarthritis. In addition, obesity is recognized as an independent risk factor for the development of chronic kidney disease (CKD)[1,2]. A reduction in body weight (BW) can improve proteinuria,[3–5] and glomerular filtration rate (GFR).[6]. Hence, emphasis has recently been placed on the importance of optimal obesity management, and the control of the fat mass. In addition, more evidence-based data demonstrating the detrimental role fat mass accumulation over time may be necessary.

Further, the predictive ability of abdominal or central obesity to identify individuals with increased health risks might be more evident.[7,8] Visceral obesity is closely associated with various metabolic and cardiovascular complications such as insulin resistance, hyperlipidemia, hypertension, and even mortality above and beyond that associated with total adiposity.[9–14] Thus, comprehensive evaluations are needed to understand the health risk implications of increased visceral fat independently from the risks associated with total or subcutaneous obesity.[15].

Although a few limited number of studies have evaluated the association between visceral adipose tissue and albuminuria[16], most analyses were conducted among diabetic patients [17,18] and displayed as a cross-sectional design.[15,16] To date, no longitudinal study has examined changes in visceral fat mass (∆VFM) over several years, and the implied health risks such as development of proteinuria. Because the purpose of diagnosing visceral obesity is to minimize future accumulation of visceral fat to reduce multiple metabolic risks,[19] in this longitudinal cohort study of the general Korean population, the effects of baseline VFM and 4-year changes in VFM (∆VFM) on proteinuria development were prospectively evaluated.

## Materials and Methods

### Study subjects

We conducted a prospective longitudinal cohort study with 2,809 individuals of the Korean general population who participated in two health screening check-ups separated by a 4-year period (2008–2012, 2009–2013). To evaluate the relationship between obesity and proteinuria development, individuals with the following criteria were excluded (n = 198); pre-existing proteinuria at the baseline exam (n = 48); previous history of urologic malignancy (n = 6); glomerulonephritis (n = 12); CKD or undergoing dialysis (n = 51); and missing data at baseline (n = 81). During the study period, 218 subjects were lost to follow-up; therefore, the study sample comprised 2,393 subjects. The study was conducted according to the Declaration of Helsinki. Written informed consent was obtained from each subject after a full explanation of the purpose and nature of the study. The protocol was approved by the institutional review board/ethics committee of Hallym University Sacred Heart Hospital, Anyang, Korea.

### Data collection and proteinuria evaluation

The subjects were asked to describe their smoking habits, alcohol consumption, physical activity, drug history, marital status, socioeconomic status, diet, and previous medical conditions such as diabetes, hypertension, cardiovascular disease, gout, and malignancy. Blood samples were collected in the morning after an overnight fast. Serum hemoglobin, glucose, total cholesterol, triglycerides (TG), low-density lipoprotein (LDL), high-density lipoprotein (HDL), albumin, and alkaline phosphatase levels were measured. Serum high-sensitivity C-reactive protein (hs-CRP) levels were also checked. For measurement of renal function, eGFR was calculated using the CKD Epidemiology Collaboration equations. To detect proteinuria or hematuria, dipstick urinalysis was performed using spontaneously voided fresh urine that was analyzed within a few minutes after collection. Urinalysis was not performed in subjects who were menstruating or exhibiting symptoms of urinary tract infection or vaginal discharge. The results were interpreted by one physician and were scored as (−) when no staining was observed, (±) when weak staining was observed, and 1+, 2+, 3+, or 4+ when mild-to-strong staining was observed. Proteinuria was defined as 1+ or greater.

### Adiposity and fat mass evaluation

Two trained medical staff members performed anthropometric measurements and body composition analysis following a strict protocol, as we previously reported. Waist circumference (WC) and hip circumference (HC) were measured and the body mass index (BMI) was calculated as the individual’s weight (kg) divided by height squared (m^2^). Obesity was defined as BMI ≥25 kg/m^2^. Body composition data were obtained using a multifrequency bioelectrical impedance analyzer (Zeus 9.9 PLUS; Jawon Medical, Korea). The subjects were asked to avoid eating or drinking anything except water, and the test was performed after full voiding. Using the tetrapolar electrode method (electrodes are located on both hands, the soles of both feet, and both ankles; frequency: 1, 5, 50, 250, 550, and 1000 kHz; current: 360 uA), the machine sent a minute electric current and measured the body composition using personal data that had already been saved (height, weight, sex, age, and newly calculated body impedance). The data about fat mass (FM), percentage of body fat (PBF, %), and VFM (kg) were obtained. ∆VFM during 4 years were calculated as the absolute difference of VFM at baseline and follow-up. Subjects were divided into 3 groups by gender-specific tertile of the baseline VFM and ∆VFM.

### Statistical analysis

Statistical analyses were performed using SPSS version 24.0 (SPSS Inc., Chicago, IL, USA). All variables are expressed as the mean ± SD or median with interquartile range unless otherwise indicated. The Kolmogorov–Smirnov test was used to analyze the normality of distribution, and for skewed data such as the serum hs-CRP, natural log values were used. Multiple logistic regression analysis was performed to evaluate the risk factors of proteinuria development. In adjusted model age, smoking, systolic BP, serum creatinine and hs-CRP levels were included. A p-value of <0.05 was considered statistically significant.

## Results

### Baseline Characteristics and proteinuria development

Table 1 shows the baseline gender-specific baseline characteristics of the 2393 participants. The mean age was 51.9±7.7 years, and 23.4% were men. Men were significantly older and had higher systolic and diastolic blood pressure (BP) and BMI than women. As expected, bioimpedance analysis revealed markedly higher PBF and VFM in men. Other metabolic parameters including BMI, WC, WHR, serum HDL, triglyceride, glucose, and hs-CRP levels were significantly worse in men. Baseline serum creatinine levels were also higher in men.

**Table 1:** Baseline characteristics of study participants.

	Men (n = 561)		Women (n = 1832)	
Variables	baseline	follow-up	baseline	follow-up
Age, years*	54.2 ± 7.9	58.7 ± 8.1	51.2 ± 7.5	55.8 ± 7.6
SBP, mmHg*	123.3 ± 15.0	122.1 ± 16.4	117.4 ± 15.2	117.8 ± 171
DBP, mmHg*	77.6 ± 11.3	75.4 ± 10.6	72.4 ± 11.2	67.8 ± 10.6
Pulse pressure, mmHg*	46.3 ± 11.0	48.6 ± 12.2	44.9 ± 11.4	47.4 ± 12.6
Smoking, n (%)	111 (19.8)	102 (18.2)	61 (3.3)	51 (2.7)
BMI, kg/m^2^ *	24.6 ± 2.6	24.3 ± 2.8	23.3 ± 2.8	23.0 ± 2.9
BMI >25 kg/m^2^, n (%)*	257 (45.8)	209 (37.2)	445 (24.3)	386 (21.1)
Waist circumference (cm)*	83.9 ± 9.5	82.4 ± 8.5	76.8 ± 8.0	75.7 ± 8.1
Hip circumference (cm)*	96.6 ± 5.2	93.9 ± 5.4	93.6 ± 5.4	91.4 ± 6.1
WHR*	0.87 ± 0.07	0.91 ± 0.06	0.82 ± 0.06	0.83 ± 0.05
PBF, %*	25.2 ± 4.68	24.2 ± 4.84	29.5 ± 4.37	28.8 ± 4.47
VFM (kg)*	2.46 ± 0.88	2.35 ± 0.90	2.01 ± 0.78	1.96 ± 0.78
VFM/BW (%)*	3.56 ± 0.90	3.45 ± 0.94	3.37 ± 0.93	3.34 ± 0.94
Albumin, g/dL	4.61 ± 0.25	4.60 ± 0.32	4.56 ± 0.24	4.66 ± 0.34
Creatinine, mg/dL*	0.93 ± 0.19	0.96 ± 0.40	0.75 ± 0.13	0.77 ± 0.18
eGFR, mL/min/1.73m^2^	91.0 ± 16.6	88.2 ± 15.8	92.2 ± 14.8	90.6 ± 12.9
Fasting glucose, mg/dL*	95.5 ± 22.4	101.2 ± 21.1	89.9 ± 14.9	96.5 ± 17.7
Uric acid, mg/dL*	5.21 ± 1.35	5.38 ± 1.39	4.07 ± 0.97	4.33 ± 1.04
Total cholesterol, mg/dL	193.3 ± 35.4	196.5 ± 36.6	193.5 ± 34.0	196.0 ± 32.2
HDL-cholesterol, mg/dL*	48.8 ± 11.6	55.4 ± 18.7	55.9 ± 12.5	61.5 ± 15.8
LDL-cholesterol, mg/dL	114.8 ± 32.4	110.6 ± 37.3	116.8 ± 30.7	118.5 ± 34.6
Triglyceride, mg/dL*	147.9 ± 94.9	141.5 ± 109.7	107.9 ± 78.2	112.6 ± 78.2
hs_CRP*	-2.34 ± 0.88	-2.10 ± 0.95	-2.90 ± 0.85	-2.75 ± 0.94
Proteinuria, n (%)	-	34 (6.1)	-	59 (3.2)

* p <0.001 between men and women

Over the 4-year follow-up, 1258 (52.5%) subjects experienced a decline in VFM, whereas 870 (36.4%) gained VFM. The remaining 235 (11.1%) participants experienced no changes in VFM. The median value of ∆VFM was – 0.10 (interquartile range [IQR]: -0.4, 0.2) and 0.00 (IQR: -0.2, 0.2) in men and women, respectively. The incidence of proteinuria development was 3.9% (n = 93), and was significantly higher in men than women (6.1% vs. 3.2%, p = 0.001).

### Parameters associated with development of proteinuria

Table 2 shows the clinical and biochemical parameters associated with the development of proteinuria. At baseline, increased age and various obesity-related cardiometabolic parameters including higher BP, increased WC, and higher levels of VFM and PBF were significantly associated with future proteinuria development. Serum hs-CRP levels were also higher in subjects with proteinuria development.

**Table 2:** Factors associated with proteinuria development.

	Men, proteinuria development			Women, proteinuria development		
Variables	+ (n = 34, 6.1%)	- (n = 527)	p	+ (n = 59, 3.2%)	- (n = 1776)	p
Age, years	58.9 ± 9.3	53.9 ± 7.7	< 0.001	54.5 ± 8.7	51.1 ± 7.4	0.001
SBP, mmHg	129.7 ± 15.4	122.6 ± 14.9	0.014	128.1 ± 18.6	117.1 ± 15.1	<0.001
DBP, mmHg	83.2 ± 9.2	77.6 ± 10.3	0.003	79.0 ± 15.0	72.2 ± 11.0	<0.001
Smoking, n (%)	14 (41.1)	97 (18.4)	0.084	10 (16.9)	51 (2.8)	0.031
BMI	26.8 ± 3.3	24.5 ± 2.6	<0.001	24.3 ± 3.4	23.3 ± 2.8	0.011
Creatinine (mg/dL)	1.04 ± 0.22	0.92 ± 0.19	<0.001	0.82 ± 0.21	0.74 ± 0.13	<0.001
eGFR (mL/min/1.73m^2^)	80.2 ± 19.4	91.7 ± 16.2	<0.001	84.6 ± 18.8	92.4 ± 14.6	<0.001
hs_CRP	-2.00 ± 0.91	-2.36 ± 0.91	0.067	-2.41 ± 1.15	-2.73 ± 0.084	0.015
WC	91.2 ± 9.5	83.4 ± 9.3	<0.001	80.1 ± 9.9	76.7 ± 7.9	0.001
∆ WC	1.93 ± 5.86	-1.2 ± 6.21	0.030	2.10 ± 5.78	-0.87 ± 6.02	0.001
VFM (kg)	3.38 ± 1.36	2.42 ± 0.81	<0.001	2.45 ± 1.0	2.02 ± 0.7	0.003
∆ VFM (kg)	0.07 ± 0.62	-0.11 ± 0.49	0.039	0.16 ± 0.68	-0.04 ± 0.39	<0.001
PBF (%)	27.4 ± 4.68	25.1 ± 4.67	0.005	30.1 ± 5.6	28.4 ± 4.3	0.036
∆ PBF (%)	0.01 ± 3.37	-0.70 ± 3.06	0.084	0.02 ± 3.79	-0.30 ± 2.59	0.058

In addition, obvious differences were found in the changes of fat mass during the 4 years in subjects with and without proteinuria development. Subjects who developed proteinuria exhibited a significant increase in ∆VFM, whereas those without proteinuria development experienced a decrease in ∆VFM during the 4 years. The ∆VFM between the two groups was 0.07 ± 0.62 vs. -0.11 ± 0.49, and 0.16 ± 0.68 vs. 0.04 ± 0.39 in men and women, respectively. Similarly, subjects that developed proteinuria exhibited significantly higher ∆WC in both genders. The observed ∆PBF also showed a similar tendency but was only marginally significant.

**Fig 1:**
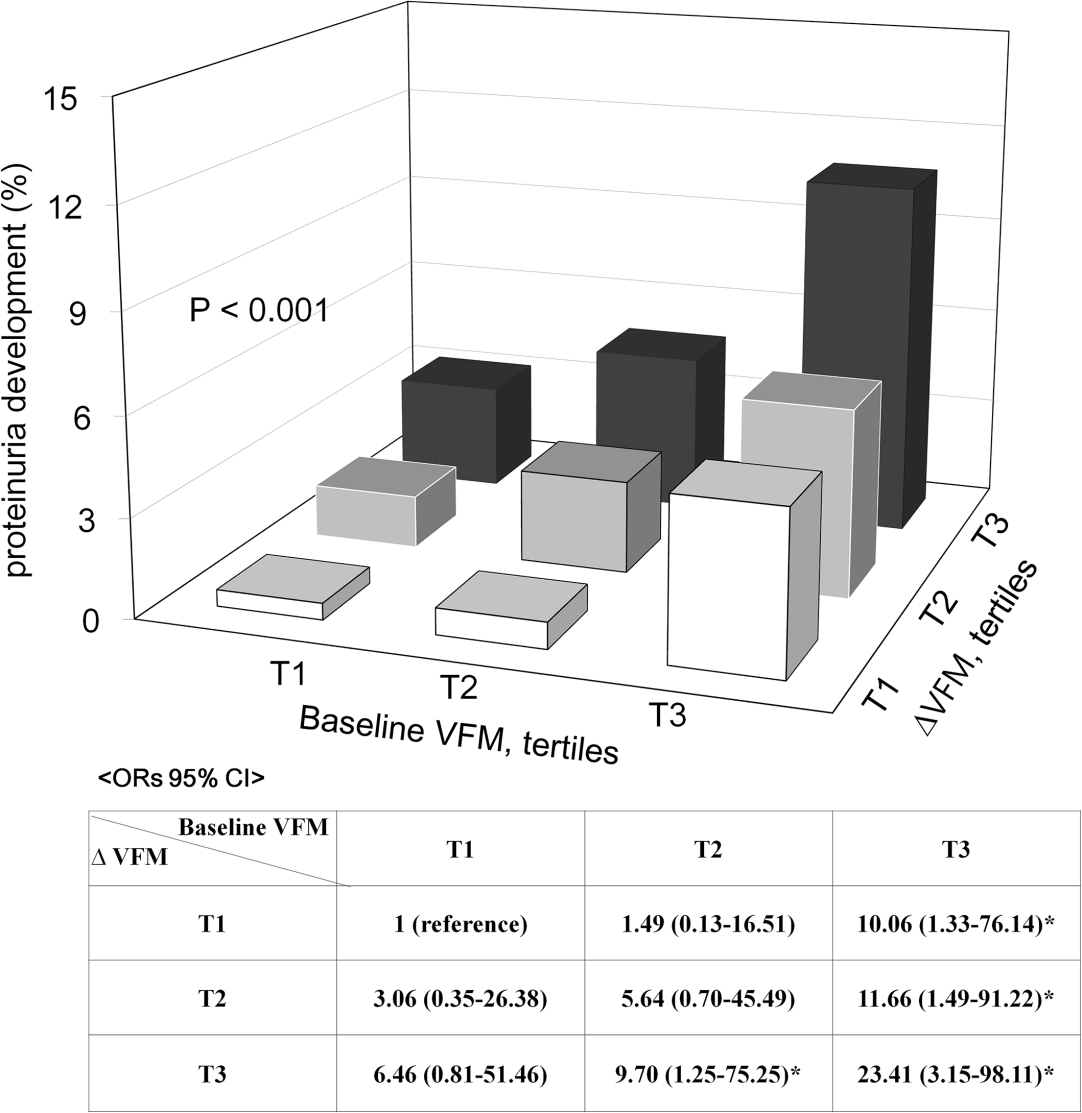
The incidence of proteinuria development according to baseline VFM and ∆VFM tertiles. Subjects in the highest baseline VFM and ∆VFM tertiles exhibited the greatest risk of proteinuria development, suggesting the additive harmful effects of the two factors.

To evaluate the prognostic value of baseline VFM and increased ∆VFM during the 4 years, participants were divided into three groups based on gender-specific tertiles of baseline VFM and ∆VFM, and the incidence of proteinuria development was compared (Table 3). In men, the incidence of proteinuria development was 2.7%, 4.3%, and 11.2% in the lower, middle, and upper tertiles of baseline VFM, respectively, which revealed a significant increase with a higher baseline VFM (trend p = 0.001). Similarly, the incidence were 3.8%, 4.3%, and 10.0% for the lower, middle, and upper ∆VFM tertiles, which indicated a progressive increase in proteinuria development with a higher ∆VFM (trend p = 0.031). The same pattern was observed in women, too. The incidence of proteinuria development in women was 1.6%, 2.6%, and 5.4% (trend p<0.001), and 1.9%, 2.8%, and 4.9% (trend p = 0.003) in the lower, middle, and upper tertiles of baseline VFM and ∆VFM, respectively. Subjects in the highest baseline VFM and ∆VFM tertiles had a significantly increased the risk of proteinuria development in both genders. Even after adjustment for age, smoking, systolic and diastolic BP, serum creatinine, and hs-CRP levels, each of the highest tertiles of baseline VFM (men; OR 3.43, 95% CI 1.22–9.67, women; OR 2.01, 95% CI 1.05–4.15) and ∆VFM (men; OR 2.92, 95% CI 1.22–6.99, women; OR 3.16, 95% CI 1.56–6.39) were independent predictors of proteinuria development (Table 3). Following adjustment of both parameters, both the highest baseline VFM and ∆VFM tertile were significant determinants of future proteinuria development (Table 4). In addition, subjects in the highest baseline VFM and ∆VFM tertiles exhibited the greatest risk of proteinuria development, which suggested the additive harmful effects of the two factors (Fig 1).

**Table 3:** OR for proteinuria development stratified by tertiles of baseline VFM and ∆VFM.

Parameters	Men (n = 561)				Women (n = 1832)			
	Total	Proteinuria development	Unadjusted OR (95% CI)	Adjusted* OR (95% CI)	Total	Proteinuria development	Unadjusted OR (95% CI)	Adjusted* OR (95% CI)
**Baseline VFM**								
**T1**	186	5 (2.7)	1 (reference)	1 (reference)	610	10(1.6)	1 (reference)	1 (reference)
**T2**	187	8 (4.3)	1.61 (0.52–5.01)	1.19 (0.37–3.83)	611	16 (2.6)	1.61 (0.73–3.58)	1.17 (0.52–2.64)
**T3**	188	21 (11.2)	4.55 (1.67–12.34)	3.43 (1.22–9.67)	611	33 (5.4)	3.42 (1.67–7.01)	2.01 (1.05–4.15)
**∆VFM during 4 years**								
**T1**	185	7 (3.8)	1 (reference)	1 (reference)	610	12 (1.9)	1 (reference)	1 (reference)
**T2**	186	8 (4.3)	1.03 (0.31–2.44)	1.02 (0.35–2.90)	612	17 (2.8)	1.44 (0.67–2.21)	1.85 (0.85–4.00)
**T3**	190	19 (10.0)	2.45 (1.05–5.76)	2.92 (1.22–6.99)	610	30 (4.9)	2.65 (1.34–5.23)	3.16 (1.56–6.39)

*Adjusted for age, smoking, systolic and diastolic blood pressure, serum creatinine, and hs-CRP level

**Table 4:** Comparisons of WC and VFM for predicting the risk of proteinuria development.

Variables		Multivariate analysis			
		VFM		WC	
		OR (95% CI)	P	OR (95% CI)	P
Age	1 year increase	1.06 (1.02–1.10)	0.003	1.05 (1.02–1.09)	0.005
Gender	male	1.03 (0.65–1.85)	0.422	1.16 (0.61–2.22)	0.636
Smoking	presence	1.02 (0.75–2.22)	0.545	1.03 (0.71–2.89)	0.449
SBP	1 mmHg increase	1.02 (1.01–1.04)	0.038	1.02 (1.01–1.04)	0.041
baseline creatinine	1mg/dL increase	2.67 (2.19–7.11)	0.001	3.98 (2.23–10.16)	0.002
hs-CRP	1 mg/L increase	1.23 (0.94–1.59)	0.124	1.22 (0.94–1.59)	0.135
VFM or WC	T1 (reference)	-	-	-	-
	T2	1.81 (0.81–2.41)	0.143	1.11 (0.77–1.76)	0.233
	T3	2.66 (1.44–4.94)	0.002	2.07 (0.96–4.49)	0.064
∆VFM or ∆WC	T1 (reference)	-	-	-	-
	T2	1.79 (1.01–3.53)	0.048	1.85 (0.99–3.44)	0.053
	T3	3.49 (2.01–6.06)	<0.001	2.60 (1.31–5.17)	0.006

Then, to compare the effectiveness as a predictor of proteinuria development between WC and VFM, we reanalyzed our data with WC. As a result, as expected, the highest tertile of ∆ WC was an independent predictor of proteinuria development (OR 2.60, 95% CI 1.31–5.17, p = 0.006) in multivariate analysis. However, the predictive role of highest tertile of baseline WC for proteinuria development was marginally significant (OR 2.07, 95% CI 0.96–4.49, p = 0.064) (Table 4, S1 Table).

## Discussion

This epidemiological study is the first to investigate the effects of changes in ∆VFM as well as baseline VFM on proteinuria development in the general population over a 4-year period. In this study, several key observations were made: (1) during the 4 years, the incidence of proteinuria development was 3.9%, and it was significantly higher in men compared to women. (2) Approximately half of the subjects experienced a decline in ∆VFM, whereas the others gained ∆VFM or experienced no changes in ∆VFM during the 4 years. (3) Increased ∆VFM and high baseline VFM both were important risk factors for future development of proteinuria. In addition, VFM and ∆VFM had additive effects on the development of proteinuria.

Obesity has become a global epidemic, and a parallel rise in the prevalence of obesity-associated adverse cardiometabolic complications has become apparent. The medical consequences of obesity are multi-factorial, but most effects can be alleviated by lifestyle modifications, including with exercise and a reasonable caloric restriction.[20] Hence, emphasis has recently been placed on the importance of optimal obesity management, and the control of the fat mass. In addition, more evidence-based data demonstrating the detrimental role fat mass accumulation over time may be necessary. Particularly, increased visceral fat deposition had more important clinical implications: it is regarded as a more relevant index of incremental cardiometabolic risks than subcutaneous fat or BMI.[12,14] Moreover, its association with a worse cardiometabolic profile is consistently observed in individuals with normal weight. According to a recently reported data, even in nonobese and apparently healthy young population, visceral adipose tissue and other regional adipose tissue such as epicardial adipose tissue carried significant correlations with all markers of cardiometabolic risk.[21] Accordingly, a 4-year changes in visceral adiposity and the resulting effect on proteinuria development was evaluated with 2393 members of the Korean general population in this study.

To date, several previous studies have reported the close relationship between obesity and renal injury. In a cohort of over 11,000 apparently healthy men followed for 14 years, higher baseline BMI was associated with an increased risk of incident CKD. Compared to participants with a BMI <22.7 kg/m2, those with BMI >26.6 kg/m2 had an odds ratio of 1.45 for developing CKD.[22] Additionally, in a non-diabetic Asian population, Kim et al. reported that the cross-sectional area of visceral adipose tissue measured using computed tomography was closely related to the prevalence of microalbuminuria, and that higher levels of visceral adipose tissue were associated with greater prevalence of microalbuminuria.[16] Also in the current study, increased baseline VFM was a significant risk factor for future development of proteinuria. When adjusted for age and gender, the odds ratio for developing proteinuria in the highest baseline VFM tertile was 2.84 (1.30–4.85). Even after adjustment for other potential risk factors such as BP, inflammatory status and baseline renal function, VFM consistently affected proteinuria development.

Then, the current study further investigated the clinical implication of increased ∆VFM over time. To our knowledge, this is the first report to evaluate the role of changes in VFM over several years in the development of proteinuria in the general population. According to our results, approximately half of the total population experienced a decline in ∆VFM during the 4 years. Such a change is consistent with previous findings of Insulin Resistance Atherosclerosis Study (IRAS) family study.[23] In the epidemiologic study, the 5-year accumulation of abdominal fat is greatest in young adulthood and became attenuated with age. In fact, patients over 60 years experienced decrease in visceral adipose tissue over time, and moreover, Hispanics experienced greater decline in visceral fat. As the mean age of our cohort was more older (52.7 years) and this study was conducted with Asians, about half of the subjects in our cohort seem to experience decline in ∆VFM. Nevertheless, interestingly, subjects with newly developed proteinuria exhibited a significant increase in ∆VFM. When the subjects were divided into three groups based on the gender-specific ∆VFM tertiles, the highest ∆VFM tertile increased the risk of developing proteinuria by two or three-fold in both genders. Considering that the highest ∆VFM tertile accounted for increments of ∆VFM over 0.1 kg, the accumulation of VFM might have a possible deleterious effect on proteinuria development. Similarly to our study, several data have reported the association between changes in anthropometric indices and new-onset CKD or proteinuria. The Physician’s Heart Study showed that men with a BMI increase > 10% had a significantly increased risk for CKD compared with those who maintained their BMI within 5% of their baseline BMI.[22] And, in a study of 2861 Japanese, BMI gain more than 0.33 during 1-year follow-up significantly increased the incidence of proteinuria and a low eGFR.[24] Although there are differences in definition of abdominal obesity, lengths of follow-up, and adjustment for confounders in these studies, it seems to be obvious that increase in abdominal or visceral obesity over time may have deleterious effect on long-term renal outcomes.

In addition, when the patients were stratified into nine groups based on baseline VFM and ∆VFM tertiles, the risk of developing proteinuria was the highest in individuals with the highest baseline VFM and ∆VFM tertile, which suggested the additive harmful effects of baseline VFM and gain in VFM over time. Fat accumulation–associated potential risk factors such as inflammation, oxidative stress, activation of renin-angiotensin system, and other uncontrolled metabolic disorders might mitigate the negative effects of VFM accumulation on proteinuria development.[25–28]

However, interestingly, the predictive role of the highest tertile of baseline WC for proteinuria development was marginally significant, although the highest tertile of ∆WC was a significant predictor. With these results, we thought that although both WC and VFM showed similar results, VFM might be a more effective and accurate parameters for predicting the development of proteinuria than WC. Supporting our data, several previous studies reported that visceral fat is a more accurate index predicting the cardiometabolic risk than subcutaneous fat or WC. According to the Korea NHANES study, a large number of individuals showed metabolic abnormalities despite a smaller WC, suggesting the importance of identification of these metabolically abnormal but non-obese individuals.[29] In addition, in a previous study with the Korean premenopausal women, subjects with a lower WC and higher visceral fat area (VFA) showed a similar pattern in metabolic syndrome components compared to those with a higher WC and higher VFA. They clarified that VFA rather than WC is a major determinant of metabolic syndrome risk in premenopausal women. [30] Namely, the location of visceral fat rather than the body fat volume itself can be more hazardous to health.

The current study had several limitations. First, the effects of lifestyle changes including dietary habits and exercise, on proteinuria development during the 4-year period were not considered. Although these parameters were investigated during each health check-up, the reliability of the data was questionable, and thus the parameters were not considered. Second, VFM was measured instead of visceral fat thickness or area because ultrasonography or CT was not performed during the routine health check-ups. However, difference in clinical significance in VFM and visceral fat area or thickness might be minimal. Third, in the study cohorts, the number of men was relatively smaller than the number of women. Therefore, the statistical power in men might be reduced.

## Conclusions

This is the first longitudinal study to evaluate changes in ∆VFM and the associated effects on proteinuria development over a 4-year period. The results revealed that both increased baseline VFM and greater ∆VFM over the 4-years were important risk factors for the development of proteinuria in the general population. Moreover, VFM and ∆VFM have additive effects on the potential for the development of proteinuria. Therefore, adequate education and interventions to prevent accumulation of visceral fat over time should be the major focus of preemptive strategies.

## Supporting Information

S1 Table OR for proteinuria development stratified by tertiles of baseline WC and ∆WC. (DOC)
