# Supplementary material for: Four-Year Changes in Visceral Fat Mass and the Risk of Developing Proteinuria in the General Population
Source: PLoS One. 2015 Jun 17;10(6):e0131119. doi: 10.1371/journal.pone.0131119 (PMC4471239; doi:10.1371/journal.pone.0131119)
Supplement: S1 Table — (DOC) [file pone.0131119.s001.doc]

Table A. OR for proteinuria development stratified by tertiles of baseline WC and ∆WC

| **Parameters** | **Men (n = 561)** | | | | | **Women (n = 1832)** | | | | | | | |
| --- | --- | --- | --- | --- | --- | --- | --- | --- | --- | --- | --- | --- | --- |
|  | Total | Proteinuria development | Unadjusted  OR (95% CI) | Adjusted*  OR (95% CI) | |  | Total | Proteinuria  development | | | | Unadjusted  OR (95% CI) | Adjusted*  OR (95% CI) |
| **Baseline WC** | | | | | | | | | | | | | |
| **T1** | 185 | 4 (2.2) | 1 (reference) | 1 (reference) |  | | 612 | | | 12 (1.9) | | 1 (reference) | 1 (reference) |
| **T2** | 187 | 8 (4.2) | 2.09 (0.61-7.07) | 1.55 (0.43-5.62) |  | | 611 | | | 14 (2.3) | | 1.22 (0.53-2.06) | 1.03 (0.41-1.23) |
| **T3** | 189 | 22 (11.3) | 5.75 (1.94-17.04) | 4.12 (1.28-13.24) |  | | 609 | | | 33 (5.4) | | 2.66 (1.41-5.02) | 2.08 (1.05-4.12) |
| **∆WC during 4 years** | | | | | | | | | | | | | |
| **T1** | 186 | 6 (3.2) | 1 (reference) | 1 (reference) |  | | 612 | | 11 (1.7) | | 1 (reference) | | 1 (reference) |
| **T2** | 188 | 11 (5.9) | 1.38 (0.54-3.52) | 1.69 (0.64-4.52) |  | | 611 | | 18 (2.9) | | 2.38 (0.97-3.49) | | 1.85 (0.85-4.00) |
| **T3** | 187 | 17 (9.1) | 2.70 (1.02-4.69) | 2.65 (1.01-6.95) |  | | 609 | | 30 (4.9) | | 2.39 (1.08-5.27) | | 3.13 (1.44-6.78) |
